# Adaptive Management of Flows in a Regulated River: Flow-ecology Relationships Revealed by a 26-year, Five-treatment Flow Experiment

**DOI:** 10.1007/s00267-022-01750-4

**Published:** 2022-11-30

**Authors:** Michael J. Bradford, Josh Korman, Jeff Sneep

**Affiliations:** 1 Fisheries and Oceans Canada, West Vancouver, BC Canada; 2 Ecometric Research, Vancouver, BC Canada; 3 J. Sneep and Associates, Lillooet, BC Canada

**Keywords:** Adaptive management, Regulated river, Environmental flow, Juvenile salmonids, Flow-ecology relationships

## Abstract

Adaptive management (AM) is often proposed as a means to resolve uncertainty in the management of socio-ecological systems but successful implementation of AM is rare. We report results from a 26 year, five-treatment, AM experiment designed to inform decision makers about the response of juvenile salmonids (*Oncorhynchus spp*.) to flow releases from a dam on the regulated Bridge River, British Columbia, Canada. Treatments consisted of a baseline (no dam release) and four different dam release regimes that followed a semi-natural hydrograph but varied in the magnitude of spring-summer freshet flows. We found total salmonid biomass was highest at the lowest flow release, and decreased with increasing flow, consistent with a priori predictions made by an expert solicitation process. Species-specific responses were observed that in some cases could be attributed to interactions between the flow regime and life history. The relationship between juvenile biomass and flow resulting from the experiment can inform decisions on water management for this river. The documentation of successful AM experiments is sorely needed to allow for reflection on the circumstances when AM is likely to deliver desirable outcomes, and to improve other decision processes that require fewer resources and less time to implement.

## Introduction

Adaptive management (AM) was developed in the late 1970s as an approach for the management of complex ecological and social systems (Holling 1978). As originally conceived, AM uses deliberate experimentation to learn about the relation between management policies and environmental or social outcomes. It is based on the premise that under certain circumstances, active experimentation and monitoring can be an efficient means to overcome uncertainty about management policies (Failing et al. 2004; Murray et al. 2015). Over the years, AM has come to include not only active experimental management, but any form of feedback from management actions that allow for learning. A typical AM cycle involves (1) setting objectives, (2) using ecosystem models or other tools to predict responses to policy alternatives and to design an experimental plan, (3) implementing the plan and monitoring responses, and (4) reporting on results and using findings to modify the experiment or inform decision making (Williams and Brown 2014).

Despite the expected benefits of the AM approach of “learning by doing”, there are relatively few reported cases where experimental AM has been conceived, implemented, and reported on (Westgate et al. 2013). The AM cycle is most likely interrupted in the implementation phase, as the scale and scope of an ecosystem-level adaptive management program can challenge human and financial resources of the participants and organizations involved (Walters 1997; McFadden et al. 2011). Nonetheless, even in cases where AM is not implemented, the planning stages are often considered valuable as a mechanism for structured thinking and analysis, and enhanced participation by affected parties (Gregory et al. 2012).

Adaptive management appears to be particularly useful in the context of management of aquatic ecosystems where human demand for water for hydropower, domestic and industrial use, or irrigation-based agriculture can compete with needs for ecosystem services (Webb et al. 2017). Increasing pressure to allocate water for human use magnifies the requirement for managers to be able to accurately describe benefits that will arise from ascribing water for environmental needs. In the case of rivers regulated for human use, a large suite of models and processes have been developed to evaluate the relation between environmental water allocations and ecological values (Anderson et al. 2006; Milner et al. 2012). Most approaches are untested, however (Castleberry et al. 1996; Davies et al. 2014). As an alternative to relying on model predictions, an evidence-based approach using case histories can also be used to inform management (Gillson et al. 2019). But in the case of river flow management, it has proven difficult to establish empirically-based generalizable relations between flow and ecological values, as site-specific factors often overwhelm expected responses derived from general principles of river science or summaries of case-history data (Poff and Zimmerman 2010).

Consequently, AM has enjoyed widespread application in settings where flows from dams or other structures can be manipulated to establish the relation between flow and ecological processes or values (Konrad et al. 2011; Olden et al. 2014). Many successful AM trial experiments have evaluated the effect of pulse or flood *flows*, or other specific features of the flow regime designed to replace some of the function of natural flows that may be lost due to river regulation (Gillespie et al. 2015). Goals for these experiments include the transport of sediment (Melis et al. 2015), resetting benthic communities (Robinson et al. 2018), establishment of riparian habitats (Rood et al. 2005), and stimulation of fish behaviours such as movements and reproduction (King et al. 2010).

In addition to pulse flows, water managers also need information on the relation between the annual flow *regime* and environmental responses, as changing the annual flow regime may have significant implications for the availability of water for other uses. AM has been used to inform components of a regulated flow regime, such as increased minimum flows to restore habitats and ecosystems affected by flow diversions (Travnichek et al. 1995; Sabaton et al. 2008; Lamouroux and Olivier 2015). Most involve the application and monitoring of a single treatment or policy alternative for minimum flows. Such trials can be informative, particularly when they are spatially replicated (Hocking et al. 2021), but are insufficient to define the relationship between a flow regime and environmental value for an individual river. A range of flows are required to understand the shape of that relation to allow for an analysis of potential for tradeoffs among competing water uses, particularly if the relation is non-linear (Rosenfeld 2017). Sometimes, natural variation in flow is used to inform the establishment of environmental flow needs (Webb et al. 2017) and while these investigations can be useful to reveal linkages between flow and ecological response, they may not capture the range of effects that a dam and its operations may have on river ecosystems (Konrad et al. 2011; McManamay et al. 2016).

Here we report results of a five treatment, 26-year, experiment designed to empirically establish the relationship between flow regime and ecological values for the regulated lower Bridge River, British Columbia, Canada. After the baseline period, the initial experimental treatment involved releasing water from a diversion dam to re-establish flows in a previously dry reach and increase flows in reaches further downstream that previously only received natural tributary inflows (Decker et al. 2008; Bradford et al. 2011). We now report results from subsequent treatments, and create flow-ecology relationships for this river, using abundance and biomass of juvenile salmonids (*Oncorhynchus spp*.) as our primary ecological indicators. We believe this to be the most extensive attempt to experimentally establish a flow regime-ecology relation for a regulated river, and is one of the few successfully completed long-term multi-treatment adaptive management experiment of any type (Westgate et al. 2013).

## Bridge River Flow Experiment

**Fig. 1:**
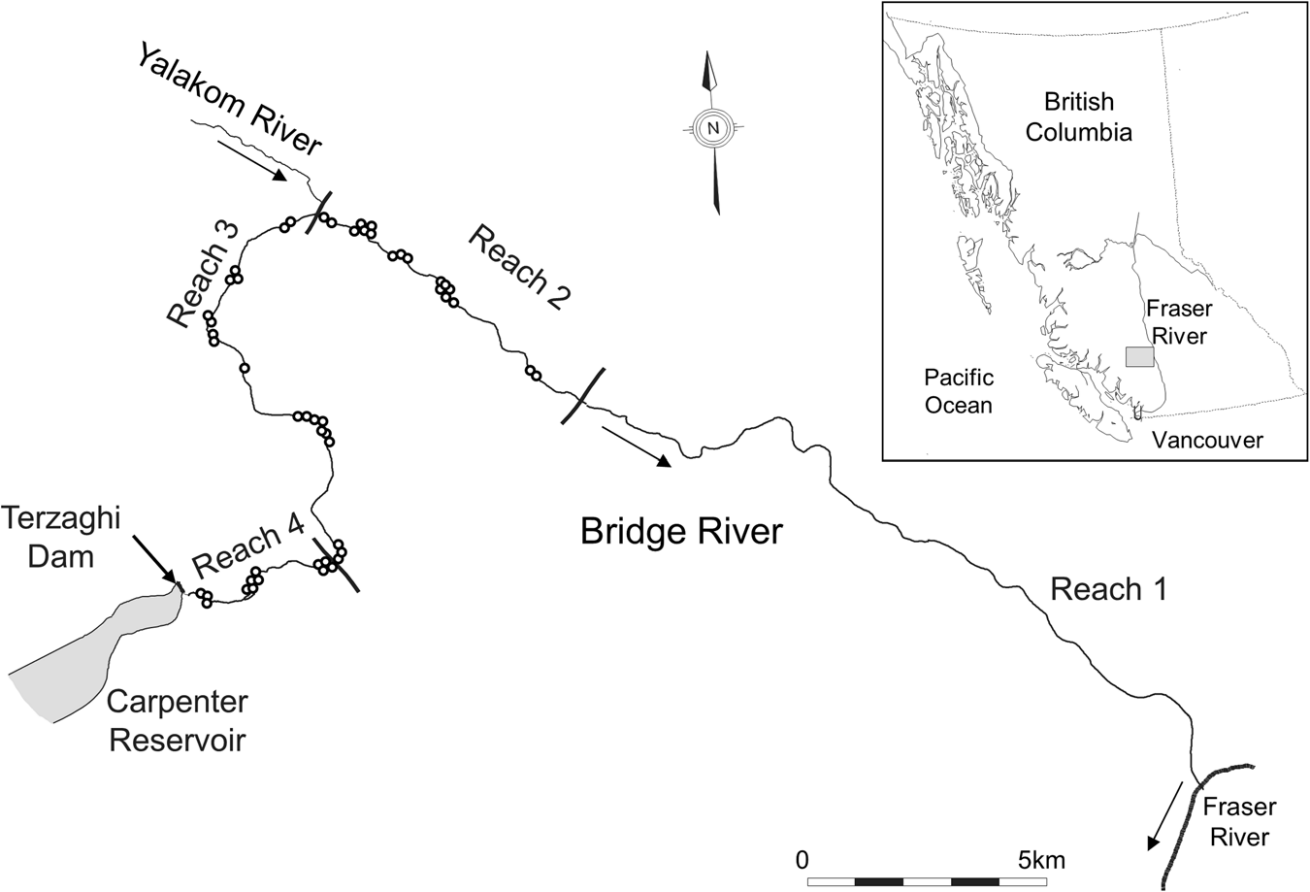
Map of the Bridge River, showing the location of the study reaches and the Yalakom River, the largest tributary. Arrows indicate the direction of flow; points are the location of the electrofishing sites

The context for this experiment is described elsewhere (Failing et al. 2004; Bradford et al. 2011; Bradford 2020) and will only be summarized here. Prior to hydroelectric development in the watershed, the lower Bridge River was a large river (mean annual discharge of 100 m^3^ ∙ s^−1^; peak flows in excess of 900 m^3^ ∙ s^−1^) that drained an area of glaciers in the Coast Range of British Columbia (Fig. 1). Like many rivers in western North America the Bridge River supported a number of Pacific salmon and trout (*Oncorhynchus spp*.) populations that are important components of both ecosystems and human social and economic systems (Healey 2009). The lower Bridge River passes through a deep canyon all the way to the Fraser River and was unlikely to have been productive habitat for the various species of salmon or trout that used the watershed. Local and traditional knowledge suggests adult salmon migrated through the canyon and spawned in lower gradient reaches of the mainstem and tributaries located further upstream.

In the mid-20th century, the Bridge River watershed was developed for hydroelectric power production. Terzaghi Dam was constructed in the canyon, 42 km upstream from the confluence with the Fraser River. The dam created a large storage impoundment that captured flows from the upper watershed. All inflows were diverted to the adjacent Seton River watershed for power generation. Downstream of the dam, the river channel was dewatered for over 3 km until groundwater and small tributaries provided sufficient inflow for continuous flows. Mean annual flow 10 km downstream of the dam was 1.2 m^3^ ∙ s^−1^, about 1% of the historical flow. The Yalakom River, a major unregulated tributary that has mean annual discharge of 4.6 m^3^ ∙ s^−1^, joins the Bridge River 15 km downstream of the dam.

In the 1990s agreement was reached between regulatory agencies and BC Hydro, the operator of the facility, to release water into the previously dry reach of the river using a gate at the base of the dam. Juvenile salmon biomass was initially chosen as the primary ecological indicator for the Bridge River, as salmon production was considered an important ecosystem service and it was assumed to serve as a proxy for habitat quality and other aspects of river health. It was assumed that the abundance of juveniles would not be recruitment limited, and even at low spawner abundances there would be sufficient juveniles to seed all available habitats (Bradford et al. 2011).

During the planning of the Bridge River flow experiment, two alternatives for the effect of flow on salmonid biomass were advanced as a result of uncertainty about how increased flow would affect conditions for juvenile fish in the river (Failing et al. 2004). The “high-good” hypothesis proposed that the increase in wetted area resulting from restoring flow to the previously dry reach, and increasing the size of the river in more downstream reaches, would result in proportional benefits for juvenile salmon. In contrast, the “low-good” hypothesis was informed by observations and modelling on the negative effects of increased flow on physical habitat conditions, and predicted that losses in habitat quality would more than counterbalance the increased wetted area, resulting in a decline in biomass.

An expert elicitation process was used to estimate uncertainties for these hypotheses to inform a decision analysis on whether a multi-treatment AM was appropriate to resolve some of the uncertainty (Failing et al. 2004). Experts showed preference for the “low-good” hypothesis that larger flow release would result in conditions less suitable for juvenile salmon. However, the uncertainty about the true state of nature was large enough to warrant the use of a flow release experiment to inform decision making (Failing et al. 2004).

The original experimental design was a series of 4-year trials beginning in 1996, based on a sequence of 0 (baseline), 3, 1 and 6 m^3^ ∙ s^−1^ annualized water budgets, with a monthly release pattern that resembled the natural hydrograph for the region (Failing et al. 2004). However, the implementation deviated from the initial design and consisted of 4 years of baseline conditions, 11 years with a flow release based on a 3 m^3^ ∙ s^−1^ annual water budget, and 5 years of a 6 m^3^ ∙ s^−1^ water budget (Table 1). In 2016, unexpected issues with the storage and generation infrastructure resulted in a need to release significantly more water into the lower Bridge River, for a period of 3 years. This unplanned event was opportunistically turned into a fourth trial that consisted of very high summer flows. In 2019, flows were restored to the 6 m^3^·s^−1^ water budget, although some higher flows occurred as a result of the infrastructure needs. We refer to these years (2019–2021) as a separate trial although the flows are similar to those of the earlier 6 m^3^ ∙ s^−1^ trial (Table 1).

**Table 1:** Summary of flow trials. Water budget is the average annualized flow release (m^3^s^−1^) from Terzaghi Dam, summer flow is the average flow 10 km downstream from the dam in June and July, and winter flow is the October–March flow at the same location

Trial	Years	Water budget	Summer flow	Winter flow
0	1996–2000	0	2.3 (1.0)	0.26 (0.01)
1	2000–2011	3	5.9 (0.3)	2.5 (0.01)
2	2012–2015	6	17.9 (0.3	2.7 (0.31)
3	2016–2018	~20	74.6 (7.0)	2.2 (0.03)
4	2019–2021	6	20.3 (3.3)	2.3 (0.02)

Standard errors calculated from annual values are indicated in parentheses

Bradford et al. (2011) compared results from Trial 1 to the baseline period using results from 1996–2008. The newly re-watered reach immediately downstream of the dam was quickly colonized by salmon and trout and was productive (Decker et al. 2008), but there was only a slight increase in total juvenile salmonid abundance further downstream where the dam releases augmented natural inflows. Declines in juvenile Chinook salmon (*O. tshawytscha*) abundance, an important ecosystem component for First Nations (Failing et al. 2013), were observed. It was hypothesized that increases in river water temperatures in fall and winter from the dam releases led to faster development of eggs and larvae deposited in the spawning gravels. As a result, fry earlier emergence from spawning beds was earlier, which in turn led to either poor survival, or the migration of juveniles from the river (Bradford et al. 2011).

**Fig. 2:**
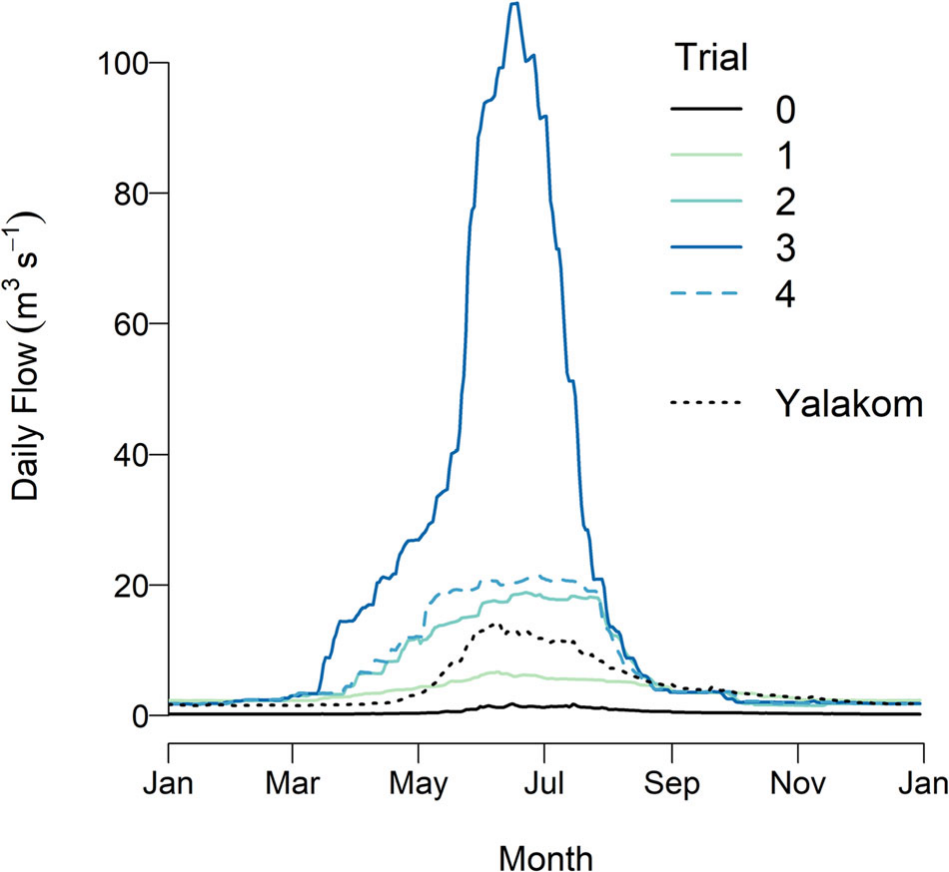
Average daily flows in Reach 3 for each flow trial. Data for 1997 (Trial 0) are excluded as there was a forced spill in late summer of that year. Also shown are the average (1996–2019) daily flows for the unregulated Yalakom River that drains into the Bridge River just upstream of Reach 2

Based on findings from Trial 1, some modifications of the next flow trial were deemed prudent to reduce risk to Chinook salmon. A water release schedule based on the 6 m^3^ ∙ s^−1^ annual water budget was developed for Trial 2, but rather than simply doubling the monthly flows from the previous trial, releases in the fall and winter months were slightly reduced during the period of Chinook salmon spawning and incubation. Reduced water releases were expected to cool more rapidly in the fall months as they flowed downstream, slowing Chinook salmon egg and larval development rates. The modified regime resulted in proportionally larger flows being released during the spring and summer (Table 1, Fig. 2). Similarly, for Trials 3 and 4, dam releases were reduced in late summer so that fall and winter flows were similar to the previous treatments. This design also minimized the effects of flow variation on our fish sampling program, which was conducted in late September. As a result of reshaping the annual hydrograph, average summer discharge (June–July) 10 km downstream from the dam varied widely among trials, creating a large contrast in flow during the spring-summer growing season (Table 1).

## Methods

### Field Methods

The lower Bridge River was divided into four reaches. Reach 1 extends 18.9 km upstream from the Fraser River, but was not sampled during the experiment due to poor access. Reach 2 is 7 km long and the upstream limit is the confluence with the Yalakom River. Reach 1 and 2 are similar in nature, being constrained by canyon walls with flows after dam closure mainly supplied by the Yalakom River. Reach 3 (11.9 km long), located upstream of the Yalakom River, was wetted by groundwater and small tributary inflows prior to the flow release. Finally, Reach 4 (3.2 km long) is immediately downstream of the dam and was without continuous flow during the baseline period.

Dataloggers were used to monitor water temperatures. Discharge in each reach was estimated using a combination of dam releases, discharge estimates from the Water Survey of Canada station (08ME025) in the Yalakom River, and estimates of inflow from small tributaries inferred from watershed area. Habitat surveys were conducted across a range of flows, and wetted width, mid-channel depth and mean velocity measurements were taken at 2 or more locations in each habitat unit (e.g., cascades, runs, riffles, pools). Depth and velocity measurements were not possible at the highest flows. Data were averaged by reach and flow trial.

Juvenile salmon abundance was estimated from depletion electrofishing conducted at 17–18 sites in Reach 2, 18–20 sites in Reach 3, and 12 sites in reach 4. Sites were initially established with a random sampling scheme stratified by habitat type. Sites remained fixed for the duration of the experiment, with the exception of minor adjustments that were required due to sediment inflows or other events that altered the nature of the site. Sampling sites were enclosed with block nets and repeated passes with a backpack electrofisher were conducted. Three passes were normally used, but an additional pass was employed if a decline in catches among passes was not observed. Each site was sampled once during September of each year when flows were similar among trials (Fig. 2). A large sample of fish from the electrofishing surveys were measured for fork length (nearest mm) and weighed (nearest 0.1 g). Length frequency analysis was used to separate age classes. Data for age-0 Chinook and coho salmon (*O. kisutch*), and age-0 and age-1 rainbow trout (*O. mykiss*) were analyzed. Anadromous steelhead (*O. mykiss*) also spawn in the Bridge River, but age-0 and age-1 juveniles could not be separated from resident rainbow trout in the field and all are referred to as rainbow trout here. These species and age classes accounted for the majority of the juvenile fish present in the river.

### Data Analysis

Abundance and biomass of juvenile salmon in each reach and year, along with associated observation errors were estimated with the hierarchical Bayesian model (HBM) described in Bradford et al. (2011). That model combined density estimates from shoreline samples with observations of the proportion of fish that were further offshore to generate a total annual population estimate for each reach. Modifications to priors were made for the current analysis to deal with low abundance in some reaches (see Appendix 1 of the Supporting Information). Abundance and biomass estimates were converted to lineal measures of density (fish·m^−1^, g·m^−1^) to account for differences in reach lengths.

To estimate the effects of flow regime on lineal density and biomass for each species and age class we used a mixed effect model,

1 $$\hat d_{r,y} = \rho _r + \phi _{f,r} + \gamma _y + \varepsilon _{r,y}$$

where $\hat d_{r,y}$ is the predicted density (or biomass) in reach *r* in year *y* in log space, ρ_r_ is a fixed effect of reach in the absence a flow release (log density when no flow was released from the dam under Trial 0), ϕ_f,r_ is a fixed effect for each flow trial *f*, by reach, γ_y_ is a random effect of year, and ε_r,y_ is a random effect for process error that varies by reach and year. Random year and process error effects are drawn from zero-centered normal distributions with estimated standard deviations σ_y_ and σ_p_, respectively (i.e., β_y_~dnorm(0, σ_y_)). For Trial 0, ρ_r_ is set to −100 for reach 4 resulting in a prediction of near zero for density or biomass when there was no flow in this reach.

There were 71 estimates of density and biomass, and their uncertainties, across all year-reach combinations over 25 separate years for age-0 data (all species), and 70 estimates for age-1 data (rainbow trout) estimated by the HBM (Appendix 2 of the Supporting Information). As the implementation of Trial 1 began at the beginning of August, 2000, age-0 data for 2000, and age-1 data for 2001 were excluded from the analysis because the effects of flow on the early life history could not be clearly ascribed to one flow regime. Both age-0 and age-1 versions of the model estimate 14 fixed effects (2 reach effects and 12 reach-flow treatment effects). A total 112 and 111 parameters are estimated when fitting the flow effect model (Eq. 1) to age-0 and age-1 data, respectively. There are more parameters estimated than observations, but the parameters are identifiable because the random year and process error effects are constrained by their prior distributions. Owing to constraints associated with these priors, the model will attempt to explain as much of the variation as possible using the fixed effects.

The likelihood of the density and biomass estimates given the predictions made by the fixed effect model was calculated by comparing estimates (d_r,y_) from the HBM and predicted log density or biomass (Eq. 1) using,

2 $$d_{r,y} \sim dnorm\left( {\hat d_{r,y},\sigma _{o_{r,y}}^{ - 2}} \right)$$

where $\sigma _{o_{r,y}}^{ - 2}$ is the inverse of the observation error variance (i.e., precision) in the log density or biomass for each reach and year. Estimates from the HBM ($d_{r,y},\,\sigma _{o_{r,y}}^{ - 2}$) are treated as observations in the mixed effects model. As some deviation between predictions and data are expected due to observation error, predictions of log density or biomass from the mixed effects model (Eq. 1) are not expected to match the values generated by the HBM perfectly. In essence, the process variance (σ_p_^2^) only needs to be large enough to explain the predictions after observation error has been accounted for via Eq. 2. The model was fit in winBUGS (Linn et al. 2000) using uninformative uniform prior distributions for all fixed effects and variance terms. To achieve adequate model convergence, as assessed by the Gelman-Rubin convergence statistic (Gelman et al. (2004), $\hat r$ ≤ 1.05), we saved every 10th MCMC sample from a total of 25,000 for each of 3 chains initialized with random starting values, after discarding the first 10,000 samples to eliminate initialization effects.

Multi-level *R*^2^ statistics (Gelman and Pardoe 2006, Recchia 2010) were used to describe fit and quantify the explanatory ability of fixed effects. At the data level, the familiar square of the Pearson correlation coefficient indicates how much of the total variation in observed log density or biomass is explained by the model, as determined by both fixed and random effects. We refer to this coefficient as the data *R*^2^. At the next level, the ratio of the variation in predicted log density or biomass across reach and year strata explained by fixed effects, relative to its total variation resulting from both fixed and random effects, is used to quantify the explanatory power of fixed effects. We refer to this coefficient as the fixed effects *R*^2^. Unstratified and stratified flow effect models were compared using the Deviance Information Criteria (DIC) which is a Bayesian analog of the Akaike Information Criteria (Spiegelhalter et al. 2002). The source code for the unstratified model is provided in Appendix 3 of the Supporting Information.

We defined the flow-ecology relation as the relation between total juvenile salmonid abundance and biomass summed over all reaches and the average June-July flow, as flow during spring-summer was the primary difference among treatments. We calculated the total annual fall abundance or biomass summed over all reaches by MCMC sample, and then averaged the sample-specific values across years within treatments. We then computed the mean and 95% credible interval for each trial across the MCMC samples.

To estimate the effect of flow on fish size we used general linear model (GLM) with log-transformed weight for each taxa (averaged by year) as the dependent variable. We included a taxa-specific intercept and log-transformed June-July flow and conspecific abundance as covariates.

## Results

**Fig. 3:**
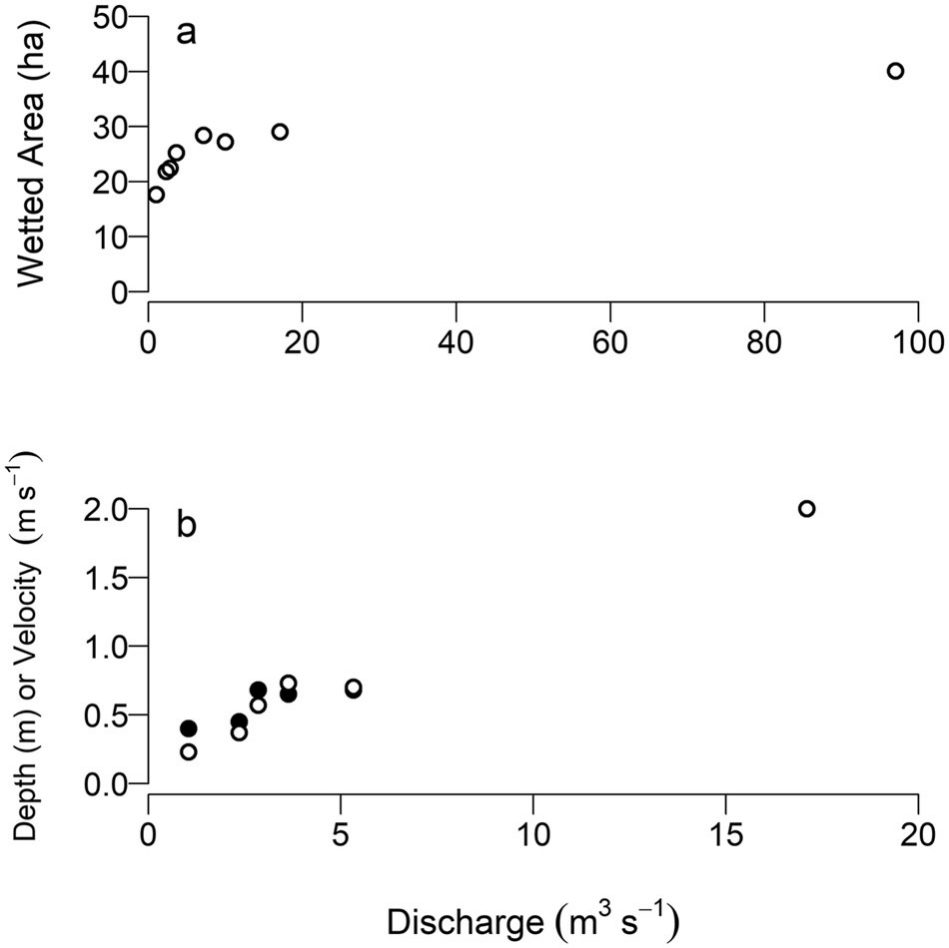
Changes in habitat conditions as a function of flow. **a** Average wetted area of Reach 3 as a function of discharge at the time of opportunistically conduced habitat surveys. **b** mid-channel mean depth (solid symbols) and mean velocity (open symbols) for Reach 3. During the highest flows it was not possible to obtain depth measurements; the highest velocity estimate is for surface water and may overestimate mean water column velocity

There was strong contrast in flow, water depth, wetted area, and velocity among treatments. The largest changes in wetted area occurred with the smallest increments in flow, as the constrained nature of the channel limited increase in wetted area at higher flows (Fig. 3). It was not possible to obtain depth or velocity measurements under the highest flows but mid-channel velocities likely exceeded 2 m ∙ s^−1^ at discharges in excess of 50 m^3^ ∙ s^−1^.

**Fig. 4:**
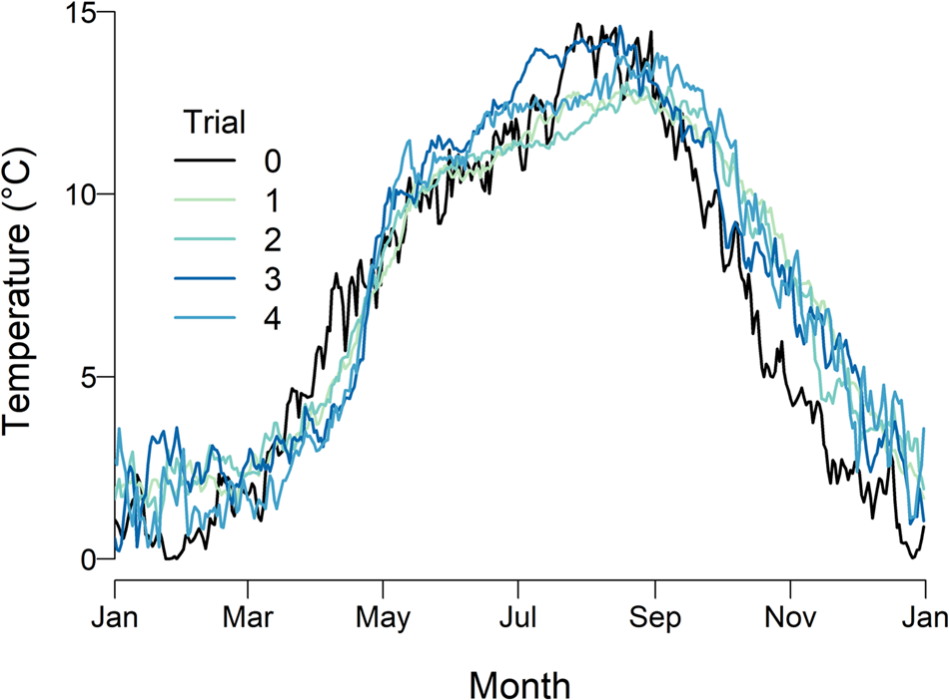
Water temperatures in the Bridge River. Shown are daily averages for Reach 3 for each flow trial

After the initiation of dam releases, water temperatures were cooler in the spring months, and warmer in the summer and fall, relative to the baseline (Fig. 4). The reduction in flow in the fall months that was implemented for Trial 2 had only a minor effect as the average October-December temperature was reduced by 0.5 °C for Trials 2–4 relative to Trial 1.

The mixed effects model fit year- and reach-specific estimates of log density and log biomass well, with *R*^2^ values between 0.86 and 0.96. *R*^*2*^ values for fixed effects, which included reach-specific constants and reach- and flow treatment-specific effects, ranged from 0.71–0.87 for density, and 0.63–0.90 for biomass.

**Fig. 5:**
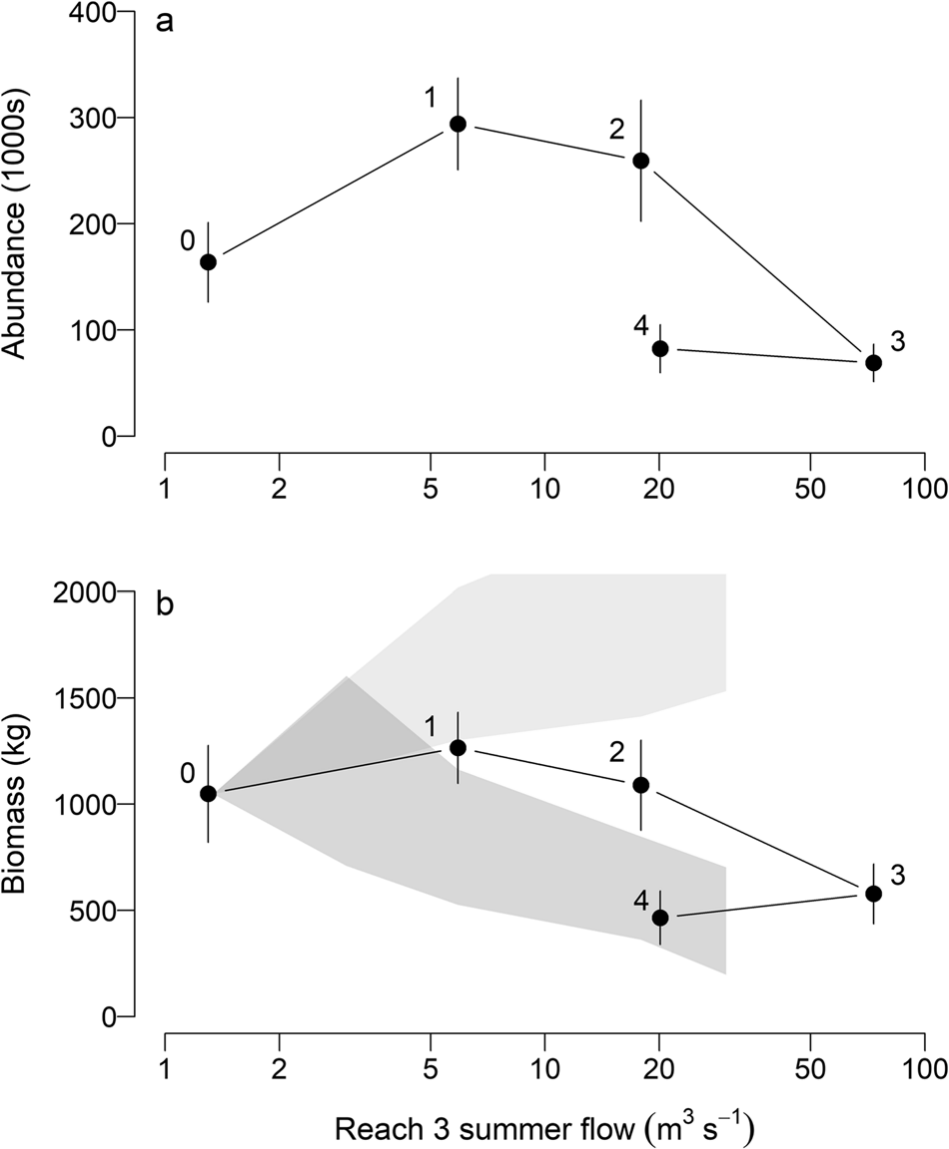
Total juvenile salmonid abundance and biomass in the lower Bridge River. Shown are medians for abundance (**a**) and biomass (**b**), with 95% credible intervals, for Reaches 2–4 as a function of the average June and July flow. Flow trials are indicated by numerals (Table 1). Shaded polygons are two competing predictions of the effects of the flow release from Failing et al. (2004). Predictions are rescaled from Failing et al. (2004) because the biomass estimate in the baseline period (Trial 0), from which the predictions were anchored, was a preliminary value that was revised during the current analysis

**Fig. 6:**
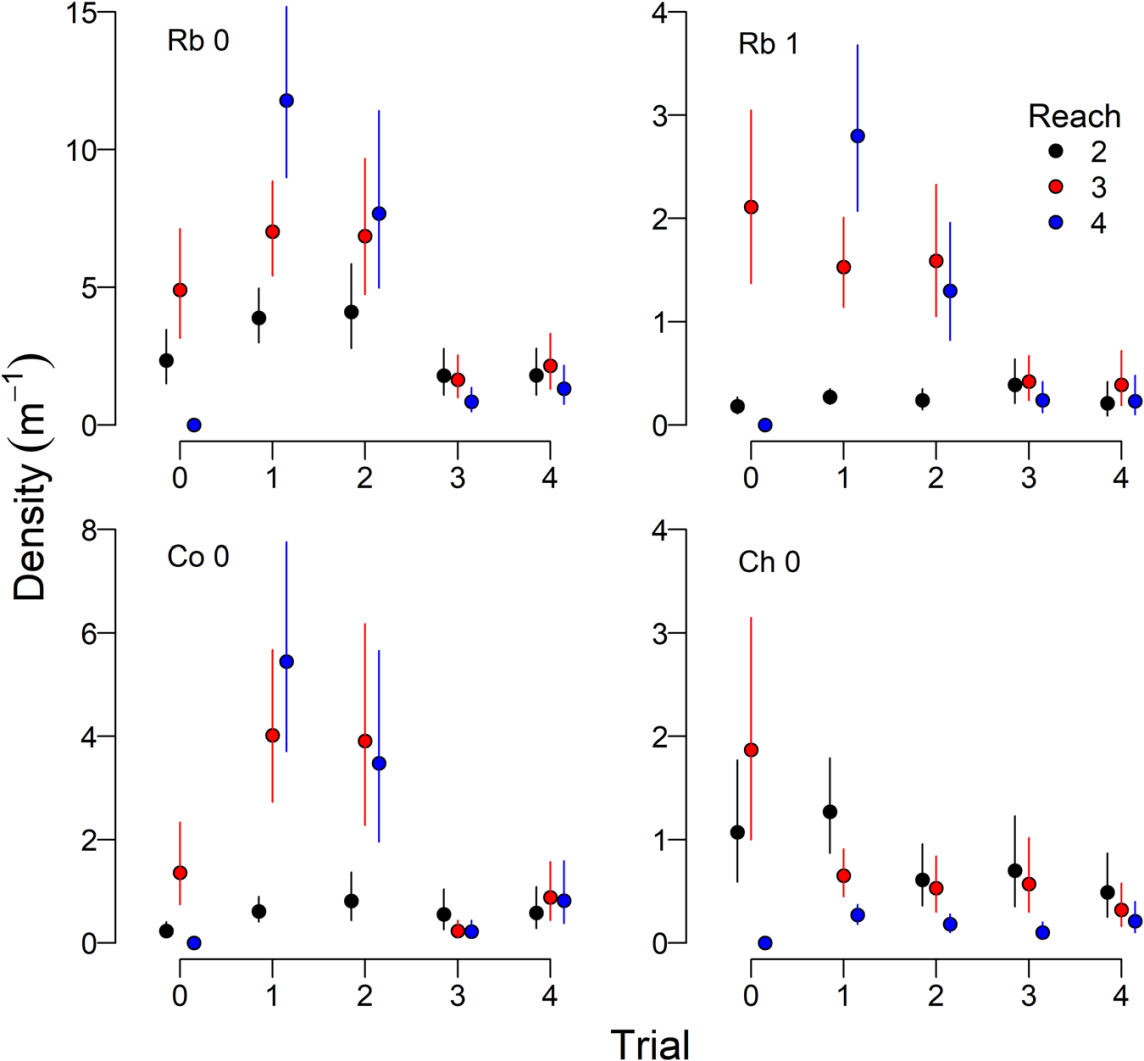
Lineal density of juvenile salmonids in the Bridge River, by reach and flow trial. Vertical lines indicate 95% credible intervals for age-0 and age-1 rainbow trout (Rb 0, Rb 1), age-0 coho (Co 0) and Chinook (Ch 0) salmon

**Fig. 7:**
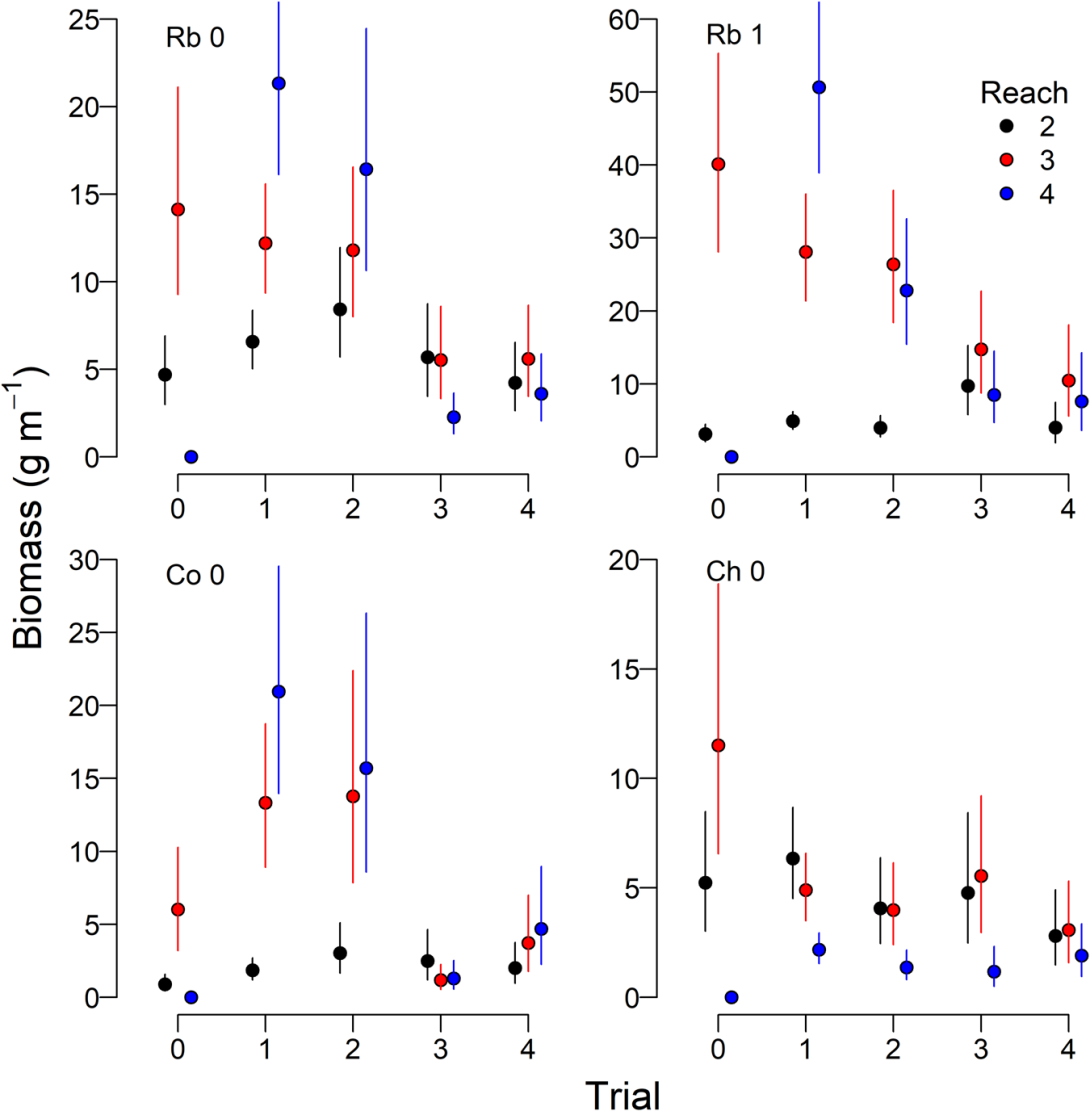
Lineal biomass of juvenile salmonids, by reach and flow trial, as in Fig. 6

The flow-ecology relations for total salmonid abundance and biomass for the study area were dome-shaped and peaked at lower summer flows (Fig. 5), and most resembled the “low-good” hypothesis that was identified by Failing et al. (2004). Rainbow trout were the most common juvenile salmonid in the river, and age-0 and age-1 groups were the main driver of the overall flow-ecology relationship for juvenile salmonid abundance and biomass, respectively (Figs. 6, 7). Although flows in Trial 4 were similar to Trial 2, total abundance and biomass did not return to levels observed in Trial 2.

We observed reach- and species-specific responses to the flow treatments. For all groups except Chinook salmon, abundances were highest during Trials 1 and 2, but declined under the high flows of Trial 3, and remained low during Trial 4 (Fig. 6). Juvenile Chinook salmon abundance declined during Trial 1, and remained low for the remainder of the experiment. Across all groups, abundances were higher in Reaches 3 and 4, and consistently low in Reach 2 during the first three trials; however, differences among reaches generally disappeared during Trials 3 and 4. Trends in reach-specific biomass estimates were similar to those for abundance (Fig. 7).

**Fig. 8:**
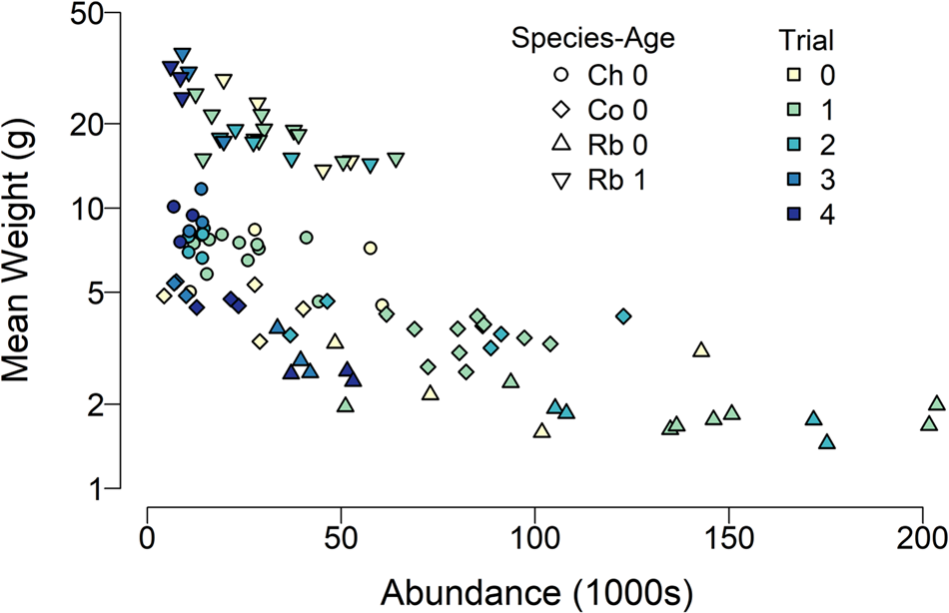
Annual mean size of juvenile salmonids in the Bridge River estimated from fall electrofishing surveys, plotted as a function of the river-wide annual estimate of abundance for each species-age group. The average sample size per point is 786, and the average standard error is 2.4% of the mean

The size of juvenile salmonids sampled in the fall decreased with increasing conspecific abundance (Fig. 8). The GLM model provided a good fit (*R*^*2*^ = 0.95), and confirmed negative effect of abundance on log-weight (coefficient = −0.002 (SE = 0.0003), *P* < 0.001) and a positive effect of summer flow (coefficient = 0.06 (SE = 0.02), *P* = 0.005) with the latter effect mainly due to the large size observed during Trial 3 (Fig. 8).

## Discussion

After five flow treatments and 26 years of monitoring, we were able to define a relationship between flow regime and our primary ecological indicator, juvenile salmonid biomass. This relation can be used for decisions about long-term flow regimes in the lower Bridge River. The results suggest once the previously dry upper reach was rewetted, additional flow provided limited benefits, and appeared to be detrimental at the highest flows. Since initiation of the experiment, the suite of values to be considered when deliberating on flows for the river has broadened (Failing et al. 2013) and the ultimate decision about a long-term flow regime for the Bridge River may be informed by other values that are more tightly linked to flow volumes than juvenile salmon biomass, such as other ecological indicators, power production, or flood and spill management (Failing et al. 2013; Capon and Capon 2017).

Flow-ecology relations are likely to be site specific and their shape will partly depend on the state of the river before experimentation. Prior to regulation, all of the Bridge River below the dam site was constrained by canyon walls and was extremely turbulent, and was unlikely to have been optimal fish habitat. Once the dam was constructed and flows were diverted, the small residual flow in wetted reaches below the dam supported juvenile salmonid abundance and biomass greater than normally observed in streams in western North America (Bradford et al. 2011), a situation that is unusual for regulated rivers where most of the water has been diverted. River environments are usually degraded by flow diversions, and in those cases positive responses by stream biota to environmental flow releases are observed (e.g., Travnichek et al. 1995; Jowett and Biggs 2006; Lamouroux and Olivier 2015). The scope for improvement in reaches that were already wetted may have been limited, and this was confirmed by early results from Trial 1 that showed little change to overall fish abundance or biomass in Reaches 2 and 3 relative to the baseline (Bradford et al. 2011). Due to similarities in habitats, we expect the response in the unsampled Reach 1 to be similar to Reach 2. It was only during the much higher flow of Trial 3 that a clear response of salmonid biomass was observed, and the dome-shaped flow-ecology relation was fully revealed. We found no evidence of thresholds (Rosenfeld 2017), perhaps because our primary indicator, total salmonid biomass, integrates effects of interactions between the various species and age groups, and local ecological conditions along the length of the river.

The response of fish populations to flow is the result of interactions between the life history and ecology of each species and age group, and changes in biotic and abiotic conditions in the river. The bed of lower Bridge River is composed of large boulders and bedrock elements (see Fig. 1 of Bradford et al. 2011), many of which are only overtopped at flows greater than about 10 m^3^ ∙ s^−1^. Complex flow patterns around these objects create low velocity areas suitable for spawning, and for feeding for juvenile fish even though the mean channel velocity between or above the boulders can be much higher (Shen and Diplas 2008; Lacey and Rennie 2012). The quantity of suitable habitat may have been relatively constant across the range of flow released during Trials 1 and 2, given the interaction between flow and channel topography, which may contribute to the small effect that flow has on juvenile abundance (Naman et al. 2020). The decline in abundance observed during Trial 3 suggests that the 4-fold increase in summer flow relative to Trial 2 overwhelmed the role that large substrate plays in generating favourable habitat conditions for small fish. The effect of high flows on habitat will depend on the shape of the river valley; in the Bridge River high velocities and unsuitable conditions occurred as a result of the constrained channel. In settings with broad floodplains or complex channel configurations, higher flows can lead to the creation of habitat that may be more suitable for young fish (Gippel and Stewardson 1998).

High flows during early life history stages of salmonids have a variety of negative effects on survival and recruitment, depending on species life history and river habitat (Seegrist and Gard 1972; Bergerot and Cattanéo 2016). High flows can result in the scouring of spawning beds and the downstream displacement of alevins and newly emerged fry (Jensen and Johnsen 1999; Cattanéo et al. 2002; Lobón‐Cerviá and Rincón 2004). Rainbow trout in our study area spawn in late May, and eggs, alevins, and newly emerged fish are present in the river during the period of maximum flow releases. Similarly, coho salmon juveniles emerge from spawning gravels in late spring and may also be vulnerable to increasing flow. For both of these taxa we observed large declines in abundance during Trial 3. Age-0 Chinook salmon declined in abundance during Trial 1, likely due to temperature effects on egg and larval development (Bradford et al. 2011), but there was little change during the high flow trials. Juvenile Chinook salmon are larger than rainbow trout and coho salmon during the summer months and may haven been able to avoid the effects of high velocities during peak flows.

In addition to effects on abundance it was also hypothesized that conditions for growth could also be negatively affected by higher flows (Failing et al. 2004). High velocities and increased turbidity can make foraging less efficient and energetically more expensive, and the highest flows could impact the density of invertebrates in the channel (McMullen and Lytle 2012). Further, it was predicted that mid-summer temperatures would be lower, potentially limiting growth (Failing et al. 2004). However, we found the size of fish captured during the fall was negatively related to intraspecific abundance, and there was evidence of an additional, positive, effect of summer flow on weight, contrary to predictions. The predicted decrease in temperature during the summer did not occur. Thus our results do not support the original hypotheses regarding the effects of flow on food production and foraging by juvenile salmon.

The patterns we observed in body size for some taxa may be due to the sequential effect of high summer flows on abundance, followed by interspecific interactions during late-summer and fall. After the peak flow in summer, differences in habitat conditions among trials diminished as dam releases were reduced to achieve salmon spawning flows of approximately 3 m^3^ s^−1^ in September. Density dependent growth may have occurred after flows receded as abundances that were set by the high flows of June and July were forced to compete for food and space in a potentially more restricted area (Dunham and Vinyard 1997). The interaction between the effects of high flows on abundance, and subsequent effects of abundance on growth has also been observed in unregulated rivers (Mundahl 2017).

The effect of the high flows on Trial 3 on juvenile salmon populations was not immediately reversed in the last trial when flows were reduced back to those of Trial 2. A number of mechanisms could have contributed to the lack of response in the first 3 years after the high flows. First, the displacement or mortality of fish associated with high flows of Trial 3 may have reduced the recruitment of spawners for the broods of Trial 4. Both coho salmon and non-anadromous rainbow trout mature at age 3 (and older for steelhead), and reduced numbers of spawners from cohorts impacted by the high flows of Trial 3 could result in fewer age-0 juveniles during Trial 4. For coho salmon this does not appear to be the case as the average parent spawner abundance in reaches 3 and 4 for Trial 4 was higher than that of Trial 3 (665 vs 364) and the observed spawner density in Trial 4 should be sufficient to fully seed the river (Bradford et al. 2000). Downstream displacement of juvenile coho salmon to other suitable habitats, and improved survival in the marine phase of the life history, may have contributed to stronger adult returns despite lower juvenile abundances in the study area. Unfortunately, adult abundance data are not available for rainbow trout and steelhead, so we cannot discount the role that reduced spawning populations are limiting juvenile population. If this is the case, additional generations may be required for population recovery.

A second hypothesis for the lack of recovery in Trial 4 is that some aspect of the physical or biotic environment was changed by high flows, which has resulted in either the poor survival of eggs, larvae or juveniles, or the downstream movement of juveniles. Our body size data do not suggest feeding conditions have deteriorated; however, there are a wide variety of other mechanisms that could lead to lower juvenile abundance (Roghair et al. 2002). Although recovery of salmonid populations from flood events is often, but not always, rapid (Detenbeck et al. 1992), additional monitoring and investigation will be required to evaluate the cause of low abundance in the post-high flow years.

### Predicting Environmental Flows

Monitoring results enabled us to adjudicate between the two hypotheses for the effects of flow on juvenile salmon biomass that were proposed during the initiation of the flow experiment (Failing et al. 2004). Juvenile biomass data were most consistent with the “low-good” hypothesis that was originally favoured by experts as juvenile salmonid biomass declined at higher flows, although the rate of decline was somewhat less than predicted (Fig. 5). The original experimental design included a flow regime based on a 1 m^3^ s^−1^ annual water budget; flows resulting from this water budget were designed to be closer to those predicted to optimize hydraulic habitat for juvenile fish (Bradford et al. 2011). This regime has not been empirically tested, and it is unclear from our results whether the release of flows lower than Trial 1 may provide additional benefits to fish.

In most settings where environmental flow determinations are needed, the use of AM will not be feasible and the process will rely on predictions made from some combination of modelling and expert opinion (Williams and Brown 2018). Predicting the response of fish populations to flow is challenging since there are many interacting factors that affect abundance (Lancaster and Downes 2010; Milner et al. 2012; Lamouroux and Olivier 2015), and it is difficult to forecast when flow will dominate other factors. Shortcomings of expert-based predictions have been revealed as experts are often overconfident of their predictive capabilities (McBride et al. 2012), and surprises (results that differ significantly from predictions) occur frequently (Pine et al. 2009; Melis et al. 2015). The experts employed by Failing et al. (2004) were experienced biologists who had conducted research on the river, and may have been sufficiently calibrated to make predictions for the Bridge River (Morgan 2014). It is worth noting that although the favoured prediction for total juvenile biomass of Failing et al. (2004) was supported by monitoring results, that was not the case for more specific predictions such as outputs from physical habitat modelling, or on the effects of flow on fish growth. Given the many challenges of implementing adaptive management, there is a need for a renewed effort in evaluating and improving expert or model-driven analysis of environmental flow regimes (Webb et al. 2017; Bradford 2020).

### Successful Experimental Management

The successful completion of AM experiments with multiple treatments are rare, as there are many barriers to successful implementation (Westgate et al. 2013). A nexus of factors resulted in the conditions necessary for the implementation of an AM experiment for flow releases. First, the Bridge River is located in a relatively remote area, and the primary values associated with the river are power production and the river ecosystem (Failing et al. 2013), which reduced constraints on the experimental design. The Bridge-Seton system is also part of a large hydroelectricity grid that allows for operational flexibility, and BC Hydro has the capacity to control water releases, permitting adherence to an experimental plan. Once BC Hydro committed to releasing water from the dam, a structured decision making (SDM) approach was implemented to evaluate policy options, including the potential for a long-term flow experiment (Failing et al. 2004; Mullen-Dalmer 2009, Gregory et al. 2006, 2012). Although local First Nations were not active participants in the initial stages of the SDM process, they viewed SDM as a key to engagement, which has been critical for maintaining the program (Failing et al. 2013).

The implementation phase of AM was facilitated by the foresight of BC Hydro biologists in establishing a robust baseline monitoring program prior to the first flow release, and in supporting other research and monitoring activities that enhanced the knowledge base and informed the experimental design. In addition, a standardized reporting system and common database was established that ensured consistent data collection across the variety of staff and organizations that have contributed to field studies. Continued engagement in the program and adaptation to a changing social environment was supported by an evolving set of values and indicators (Failing et al. 2013) and adjustments to the original experimental design, both based on interim results, and evolving relationships with partners. The significance of a small number of dedicated individuals that contributed to continuity in the program, data analysis and reporting cannot be underestimated.

Partly by design, and partly the result of fortuitous circumstances, more than two decades of experimentation and monitoring has yielded results that can inform long-term decisions about flow for the Bridge River. Although the program deviated from its original design, those departures resulted in informative outcomes. Case histories of completed AM programs like the Bridge River are needed so that the performance of AM as well as other approaches for flow management can be improved (Castleberry et al. 1996; Webb et al. 2017). It is the documentation of adaptive management programs that is most likely to lead to a more realistic understanding of the circumstances when AM will be a useful tool in the toolbox for environmental flow decision making.

## Supplementary Information

Supplementary information
