# Supplementary material for: Adaptive Management of Flows in a Regulated River: Flow-ecology Relationships Revealed by a 26-year, Five-treatment Flow Experiment
Source: Environ Manage. 2022 Nov 30;71(2):439–50. doi: 10.1007/s00267-022-01750-4 (PMC9892159; doi:10.1007/s00267-022-01750-4)
Supplement: Supplementary file 1 — Supplementary information [file 267_2022_1750_MOESM1_ESM.pdf]

Supplementary information

Bradford, MJ, Korman, J, Sneep, J.

"Adaptive management of flows in a regulated river: flow-ecology relationships revealed by a 26-year, five-treatment flow experiment."

Environmental Management

Corresponding author: Mike.Bradford@dfo-mpo.gc.ca

Appendix 1: Evaluation of effects of priors on capture probability hyper distribution parameters

To estimate juvenile salmon and steelhead abundance in this study, we initially applied the same Hierarchical Bayesian Model (HBM) used in Bradford et al. (2011) to the extended dataset (1996-2021). The model would not converge for more recent years when catches were much lower. Site-specific estimates of capture probability, which drive estimates of the hyper-distribution of capture probability, depend on the magnitude of the reduction in catches across passes.There is no information about capture probabilty at a site if no fish of a given species-age class are captured, and very little information when the catch is very low. If this pattern occurs at many sites, the hyper-distribution of capture probability will be poorly defined and more information on capture probability in the prior distribution is required to obtain reliable estimates of capture probability and abundance.

In the original application of the HBM we used an uninformative prior for the mean capture probability across sites centered at 0.5 (beta distribution with parameters beta(1,1)), and a minimally informative prior for the standard deviation in capture probabilities across sites (half-cauchy distribution with scale parameters 0 and 0.3, see Gelman 2006). To obtain more reliable estimates with sparser data from more recent years, we used a more informative prior on the mean capture probability across sites. The prior was still centered at 0.5 but we assumed more certainty about this mean level (beta(50,50)). We used a uniform prior on the precison (inverse of variance) of capture probability across sites (unif(10,500)) which constrained the maximum extent of variation in capture probability aross sites. In this appendix we compare the original year-specific estimates of capture probability hyper distribution parameters and abundance from Bradford et al. (2011) with revised estimates from the model based on the new priors for the capture probability hyper distribution parameters.

In cases where capture probability was well defined in all years because the species-age class was abundant and widely distributed across sites (e.g. age-0 rainbow trout), model estimates of the mean of

the capture probability hyper distribution were similar (Fig. A1). For other species and age classes, where there was less information in the data on capture probability, the means of the capture probability distributions from the model with the original priors were lower and less certain compared to those estimated from the revised model. This occurred because the more informative priors had a greater effect on the posterior when there was less information about parameter values from the data.

Estimates of the standard deviation of the capture probability hyper distribution, determining the extent of variability acros sites, were considerably higher and less certain based on the less informative priors used in Bradford et al. (2011), compared to the more informative ones used here (Fig. A2). Similar to the analysis of the means of the capture probability hyper distribution, differences between models were substantive for species and age classes with less information about capture probability.

The effects of revised capture probability priors on estimates of total abundance across reaches were modest (Fig. A3). Year-specific estimates of abundance and uncertainty in abundance for age-1 rainbow trout and age-0 chinook salmon based on the original model were higher than estimates based on the revised model with more informative priors. This occurred because capture probability estimates were lower in the original model with less informative priors. Abundance estimates for age-0 rainbow trout and coho salmon based on models with different priors on capture probability were similar. Estimates of flow treatment effects from the mixed effects model will largely be determined by relative differences in abundance estimates across years. Effects of prior assumptions on abundance estimates led to modest differences in the scale of abundance estimates, but did not effect relative differences in estimates over time based on the correlation in abundance estimates between models. The square of the Pearson correlation coefficient (r^2) of annual abundance estimates from the two models was 0.98 and 0.88 for age-0 and age-1 rainbow trout, 0.94 for age-0 coho salmon, and 0.92 for age-0 chinook salmon.

To better understand the effects of low catch and occupancy on estimates of abundance from the HBM, we simulated a set of catch depletions across 50 sites based on a zero-inflated log-normal

distribution of fish densities. We then applied the HBM to the simulated data and compared estimates of abundance and capture probability to the values used drive the simulation. We found that capture probability was underestimated and abundance was overestimated, and the extent of bias increased with the degree of zero-inflation in simulated fish densities. For example, when we assumed that 30% of the sample sites were unoccupied and mean density was low, abundance was overestimated by 50%. This occurred because the HBM assumes a log-normal distribution in fish density across sites and does not explicitly account for zero-inflation. When the true distribution of densities is zero-inflated, a better fit is obtained by lowering the capture probability because this increases the likelihood for sites with low or zero catch. This in turn results in an overestimate of abundance. Increasing information on capture probability in prior distributions reduces the tendency of the model to underestimate capture probability and therefore reduces the extent of positive bias in abundance. We attempted to revise the structure of the HBM to directly estimate the extent of zero-inflation, but this additional parameter was not estimable because the degree of zero-inflation and the magnitude of capture probability were confounded. That is, the model could not distinguish between cases where capture probabiltiy was high and a large fraction of sites were unoccupied, and the opposte pattern. Although directly accounting for zero-inflation in animal distributions can be accomodated in a mark-recapture framework (Conroy et al. 2008), confounding between capture probability and abundance precludes its use in studies that use depletion-based estimates.

Citations

Bradford, M.J., Higgins, P.S., Korman, J., & Sneep, J. (2011). Test of environmental flow release in a British Columbia river: does more water mean more fish? Freshwater Biology, 56, 2119-2134.

Conroy, M.J., Runge, J.P., Barker, R.J., Schofield, M.R., & Fonnesbeck, C.J. (2008). Efficient estimate of abundance for patchily distributed populations via two-phase, adaptive sampling. Ecology, 89, 3362-3370.

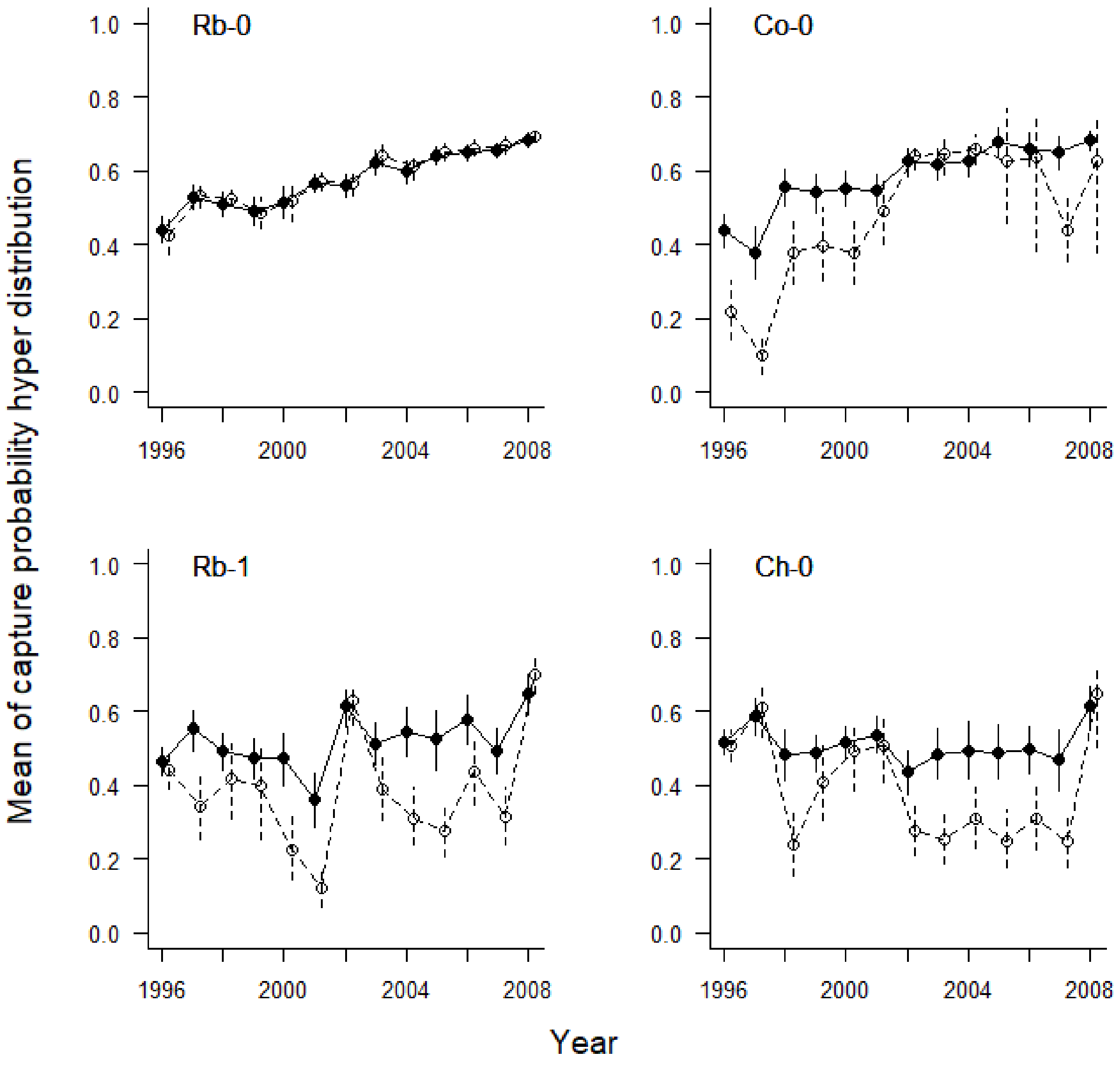

Figure A1. Annual estimates of the mean (with 90% credible interval) of the capture probability hyper distribution (distribution of capture probability across sites) based on the HBM with more restrictive priors for the capture probability hyper distribution (solid symbols and lines) and based on uninformative priors used in Bradford et al. (2011, open symbols and dashed lines).

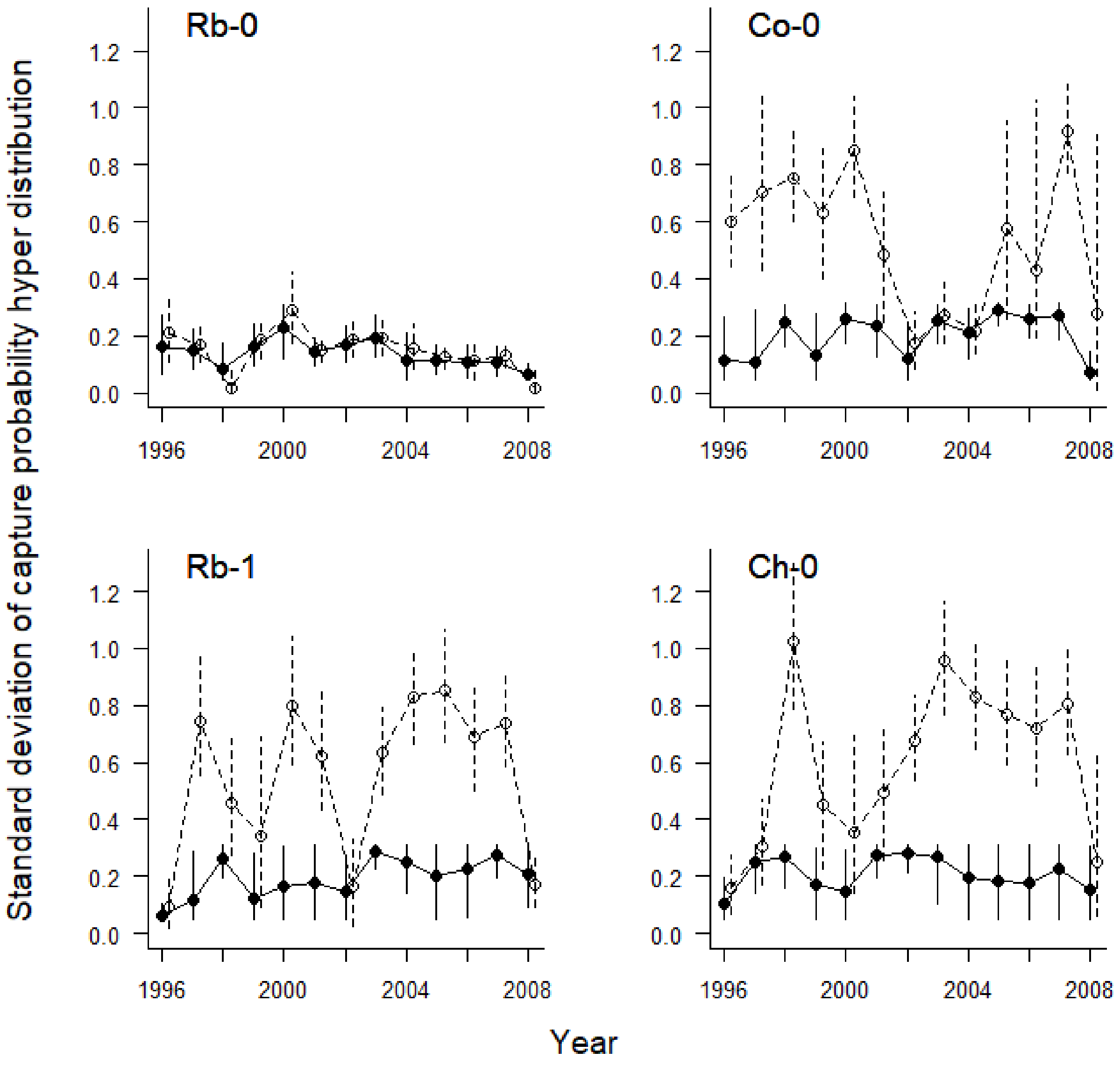

Figure A2. Annual estimates of the standard deviation in the capture probability hyper distribution based on the HBM with more restrictive priors and capture probabilities (solid symbols and lines) and based on uninformative priors used in Bradford et al. (2011, open symbols and dashed lines).

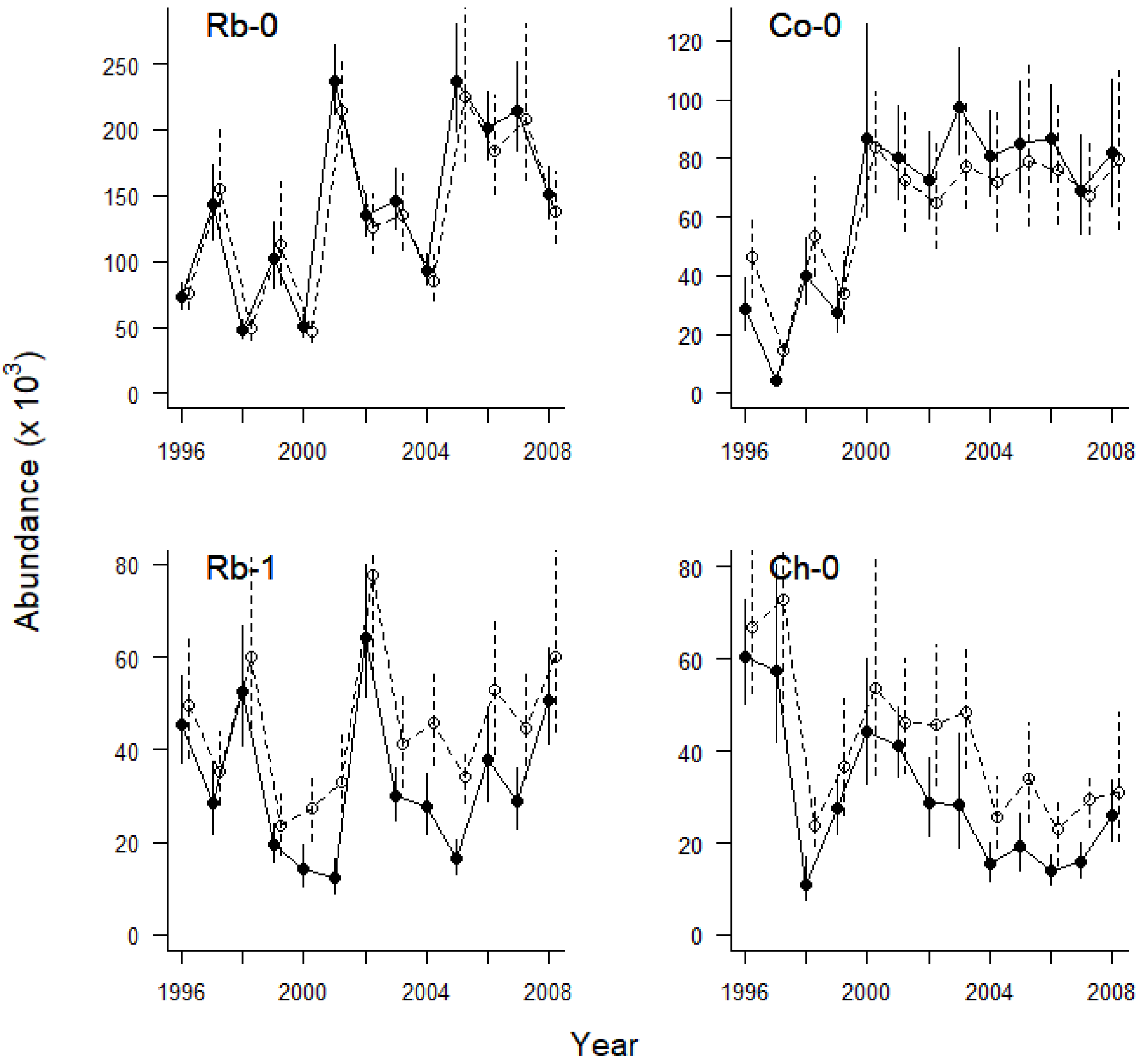

Figure A3. Annual estimates of abundance (all reaches combined) based on the HBM with more restrictive priors and capture probabilities (solid symbols and lines) and based on uninformative priors used in Bradford et al. (2011, open symbols and dashed lines).

Appendix 2. Annual estimates of abundance

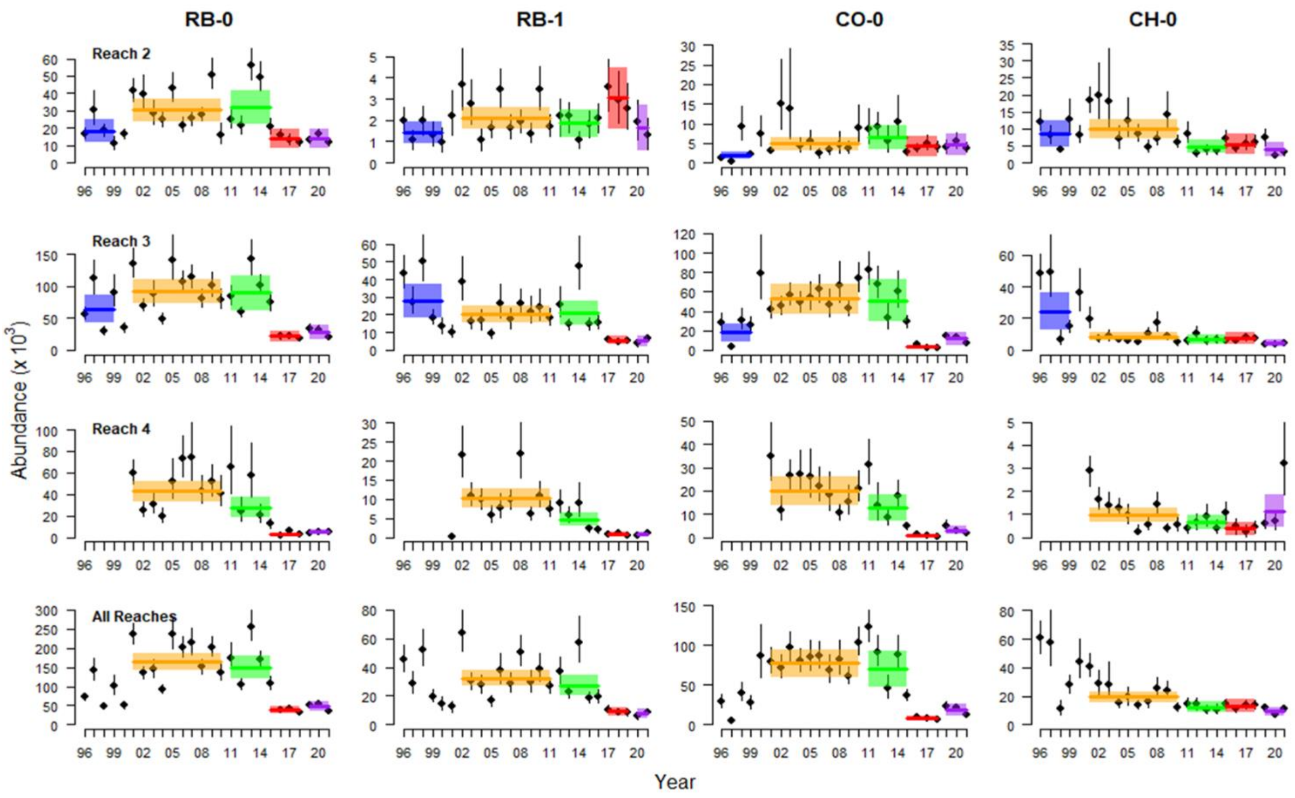

Figure A4. Annual estimates of abundance (with 95% CI) for each species/age group, by reach, and summed over all reaches. Coloured horizontal lines show the average abundance for each flow treatment estimated from the mixed effect model, the band is the 95% CI.

Appendix 3. BUGS code for mixed effects model. Text in green are comments, and bolded text represents data, and blue text represent probability distributions.

```
#Priors on fixed effects of reach and flow, year-specific random effect variance, and process variance
for(ir in 1:2) {BetaR[ir]~dnorm(0,1.0E-03)}      #fixed reach effect
BetaR[Nreaches]<- -100                           #0 fish in reach 4 at 0 cms in log space

for(ir in 1:Nreaches){
      for(itr in 1:Ntreats){
            BetaF[ir,itr]~dnorm(0,1.0E-03)       #fixed treatment effects
      }
}
yrSD~dunif(0.001,10);yrPrec<-pow(yrSD,-2)
proSD~dunif(0.001,10);  proPrec<-pow(proSD,-2)  #process error

for (i in 1:Nyrs) {BetaY[i]~dnorm(0,yrPrec)}     #random year effect

#Loop over samples (reach*year)
for(i in 1:LastTreat0){ #samples under treatment 0 that don't depend on treatment effect
      pro_dev[i]~dnorm(0,proPrec)
      pred[i] <- BetaR[Reach[i]] + BetaY[Year[i]] + pro_dev[i]    #prediction of log density or biomass
      obs_mu[i]~dnorm(pred[i],obs_prec[i])       #likelihood comparing prediction to observation
      RE[i]<-BetaY[Year[i]] + pro_dev[i]         #random effects needed for multi-level r2 calculation
}

for(i in (LastTreat0+1):Nsamps){#samples effected by treatment flows that include treatment effect BetaF
      pro_dev[i]~dnorm(0,proPrec)
      pred[i] <- BetaR[Reach[i]] + BetaF[Reach[i],Ftreat[i]] + BetaY[Year[i]] + pro_dev[i]
      obs_mu[i]~dnorm(pred[i],obs_prec[i])
      RE[i]<-BetaY[Year[i]] + pro_dev[i]
}

#Multi-level r2 computations (proportion of variation in pred[] explained by fixed effects and Pearson r2)
muRE<-sum(RE[])/Nsamps; mupred<-sum(pred[])/Nsamps; muobs_mu<-sum(obs_mu[])/Nsamps
for(i in 1:Nsamps){
      ssRE[i]<-pow(RE[i]-muRE,2)                 #sums of squares on random effects
      ssTOT[i]<-pow(pred[i]-mupred,2)            #sums of squares on fixed+random effects
      data_res[i]<-pow(obs_mu[i]-pred[i],2)      #observed - predicted (squared residual)
      data_ssTOT[i]<-pow(obs_mu[i]-muobs_mu,2)
}
```

Appendix 2. Con't.

```
FE_r2<-1.0-sum(ssRE[])/sum(ssTOT[]) #Proportion of total sums of squares explained by fixed effects
data_r2<-1.0-sum(data_res[])/sum(data_ssTOT[])  #standard Pearson r2 calculation

#Calculate density (fish/m) or biomass (g/m) (DenBio) and abundance ('000s) or total biomass (kg) (N) by
#reach and treatment (including @ 0 cms)
for(ir in 1:Nreaches){
      DenBio[ir,1]<-exp(BetaR[ir])        #0 cms
      N[ir,1]<-DenBio[ir,1]*TotLength[ir]*0.001

      for(itr in 2:(Ntreats+1)){
            DenBio[ir,itr]<-exp(BetaR[ir]+BetaF[ir,itr-1])
            N[ir,itr]<-DenBio[ir,itr]*TotLength[ir]*0.001
      }
}
for(itr in 1:(Ntreats+1)){Ntot[itr]<-sum(N[1:Nreaches,itr])} #total abundance across reaches
```
